# Novel hosts can incur fitness costs to a frugivorous insect pest

**DOI:** 10.1002/ece3.8841

**Published:** 2022-04-19

**Authors:** Timothy Lampasona, Cesar Rodriguez‐Saona, Anne L. Nielsen

**Affiliations:** ^1^ Department of Entomology Rutgers University Bridgeton New Jersey USA; ^2^ Department of Entomology Rutgers University Chatsworth New Jersey USA

**Keywords:** blueberry, *Conotrachelus nenuphar*, peach, plum curculio, preference–performance relationship

## Abstract

In phytophagous insects, adult attraction and oviposition preference for a host plant are often positively correlated with their immature fitness; however, little is known how this preference–performance relationship changes within insect populations utilizing different host plants. Here, we investigated differences in the preference and performance of two populations of a native North American frugivorous insect pest, the plum curculio (*Conotrachelus nenuphar*)—one that utilizes peaches and another that utilizes blueberries as hosts—in the Mid‐Atlantic United States. We collected *C*. *nenuphar* adult populations from peach and blueberry farms and found that they exhibited a clear preference for the odors of, as well as an ovipositional preference for, the hosts they were collected from, laying 67%–83% of their eggs in their respective collected hosts. To measure *C*. *nenuphar* larval performance, a fitness index was calculated using data on larval weights, development, and survival rate from egg to 4th instars when reared on the parent's collected and novel hosts. Larvae of *C*. *nenuphar* adults collected from peach had high fitness on peach but low fitness when reared on blueberry. In contrast, larvae from *C*. *nenuphar* adults collected in blueberry had high fitness regardless of the host on which they were reared. In this study, we show that utilizing a novel host such as blueberry incurs a fitness cost for *C*. *nenuphar* from peaches, but this cost was not observed for *C*. *nenuphar* from blueberries, indicating that the preference–performance relationship is present in the case of insects reared on peach, but insects reared on blueberry were more flexible and able to utilize either host, despite preferring blueberry.

## Graphical abstract

In this study, we use a series of experiments to test whether *Conotrachelus nenuphar*'s behavior conforms to the preference–performance hypothesis (PPH). Using insects from two hosts, peach and blueberry, we tested olfactory host preference, oviposition choice, and larval fitness between both hosts. We found that *C. nenuphar* that use peach are not able to successfully use blueberry, but *C. nenuphar* that use blueberry are able to use peach, and that choice and attraction are consistent with PPH assumptions.

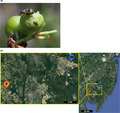

## 1 INTRODUCTION

Finding a suitable oviposition site can be a challenging task for phytophagous insects, particularly in areas with large temporal and spatial diversity of plant resources (Carrasco et al., 2015). To help narrow the search for an acceptable host, attraction and preference to hosts can be modulated by the experience of the insect. For instance, exposure to chemical cues during different stages of the insect's life can lead to a preference toward specific hosts (Anderson & Anton, 2014). Holometabolous adult insects gain experience of hosts in their immediate environment as they emerge from their pupae (Jaenike, 1983), leading them to search for similar host cues when they begin to seek feeding and oviposition sites (Baleba et al., 2020). Another mechanism is called “chemical legacy,” wherein chemical cues from the environment are stored in the hemolymph of the larvae or at the surface of the pupae and influence adult behavior. These chemical cues persist through metamorphosis and give the adult a guide when seeking oviposition sites for their own offspring (Corbet, 1985). These two mechanisms are merged under the “Natal Habitat Preference Induction” concept (Davis & Stamps, 2004). Regardless of the mechanism, the propensity to seek host environments similar to those of a natal host is broadly called “natal host fidelity” (Morris & Fellowes, 2002). Evolutionarily, natal hosts should be suitable hosts for an adult insect that has successfully developed on them. In fact, in some insects, the use of a natal host is positively correlated with improved larval performance and female‐biased offspring sex ratios (Morris & Fellowes, 2002).

Despite selective drivers pulling insects toward natal hosts, phytophagous insect populations may be pushed to utilize novel hosts. These divergences can occur for a multitude of reasons, such as a lack of preferred host material in their environment or a subset of a population developing a preference for a novel host that they can utilize more effectively than the original (Linn et al., 2003). Insects may also seek a novel host to find an enemy‐free space or to find plants that are less defended against herbivory (Linn et al., 2003). If the novel host proves to be effective for immature development, it may be utilized again by successive generations using the same selective mechanisms mentioned above (Gripenberg et al., 2010). As a result, it is hypothesized that insects should oviposit preferentially on hosts that provide a fitness benefit for their offspring, the so‐called “preference–performance hypothesis” (PPH) (Clark et al., 2011; Gripenberg et al., 2010).

The PPH has been confirmed for diverse insect taxa, including aquatic species (Bovill et al., 2013), parasitoids (Monticelli et al., 2019), and phytophagous insects, for which a female's oviposition choice is often critical for larval fitness (Gripenberg et al., 2010). Particularly, larvae that feed internally in plants are highly reliant on their mothers’ oviposition choices for survival, and as such, hosts that incur larval fitness costs may be avoided by females during oviposition site selection (Mayhew, 1998). Hence, the driving player in host selection is the female, particularly in holometabolous insects (Schoonhoven et al., 2005). A method for testing whether PPH is applicable for an insect species is to measure larval fitness on different hosts. Larval fitness refers to overall fitness when reared on a specific host, which is often measured using indices based on proxies such as larval weight, development time (Burrack & Zalom, 2008), and survival rate (Craig et al., 1989). For example, more suitable hosts should incur less fitness costs to the larvae and, in turn, hosts that incur greater larval fitness costs should be avoided by females (Baleba et al., 2020). However, two meta‐analyses suggest that the PPH is not universal (Clark et al., 2011; Gripenberg et al., 2010), with some insects showing no preference related to larval fitness and some even choosing inferior hosts when maternal feeding preferences are at odds with the offspring's needs (Clark et al., 2011; Diepenbrock et al., 2016). Based on the PPH, in this study, we tested two hypotheses, as follows: (1) that adults of a frugivorous insect pest would exhibit host fidelity by choosing the odors of, and preferring to oviposit on, the host they were collected from, and (2) that offspring fitness would also be higher on the host from which the previous generation came from.

**FIGURE 1:**
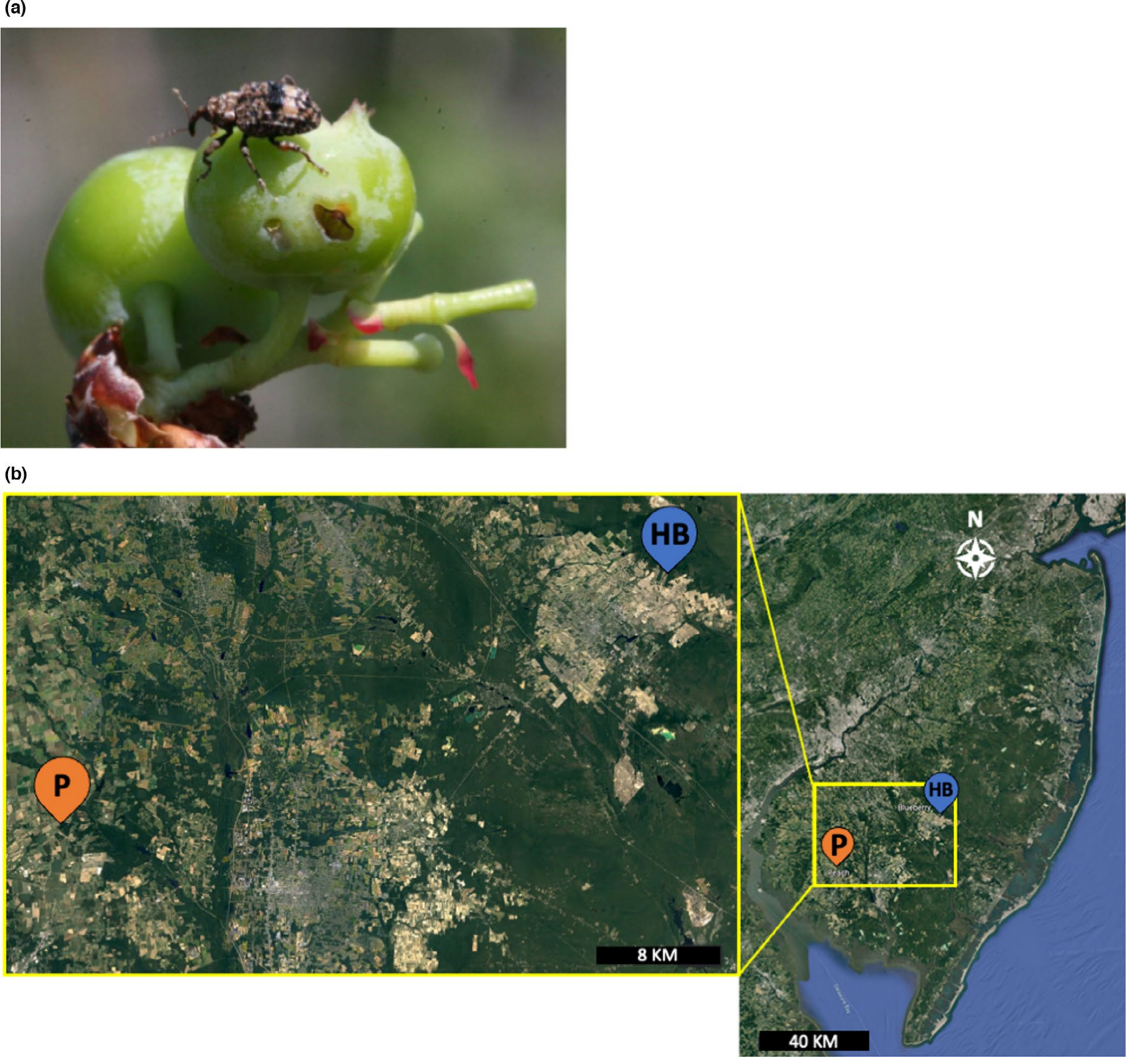
(a) Plum curculio, *Conotrachelus nenuphar* Herbst (Coleoptera: Curculionidae), adult and oviposition scar on blueberries. Photo credit: Dean Polk (Rutgers University). (b) Location of the peach (P) and highbush blueberry (HB) farms sampled for *C*. *nenuphar* adults in southern New Jersey, USA (right panel). Left panel shows a more detailed map of the locations of each of the farms. *Source*: Google Earth

As our study system, we used the plum curculio, *Conotrachelus nenuphar* Herbst (Coleoptera: Curculionidae) (Figure 1a). In the Mid‐Atlantic Region of the United States, overwintered *C*. *nenuphar* adults move into peaches and blueberries in early spring, starting in late April through early June (Lampasona et al., 2020). Once inside the crop, they aggregate, mate, and feed on the leaves and flowers until fruit set, when females begin ovipositing in the young fruit (Vincent et al., 1999). Eggs hatch within one week, and larvae feed for approximately 16 days over four instars (Crandall, 1905). Feeding by the larvae causes fruit to drop early, and once fully mature, the larvae will exit the fruit and burrow into the soil to pupate. Next‐generation adults will exit the soil and feed on nearby host plants.

*Conotrachelus nenuphar*is considered a significant pest of tree fruit across the eastern United States; however, it is only considered a key pest of blueberry in the much narrower Mid‐Atlantic Region (Lampasona et al., 2020). There is historical evidence of *C*. *nenuphar* utilizing wild Ericaceous hosts before blueberry domestication, which occurred in the last century, and it is likely that this native host was used throughout the evolutionary history of this insect (Quaintance & Jenne, 1912). However, studies on the oviposition preference of *C*. *nenuphar* indicate that they generally prefer to oviposit in stone fruit, and of those, they prefer the non‐native Japanese plum (Jenkins et al., 2006; Leskey & Wright, 2007). In fact, the preferred hosts of *C*. *nenuphar* are introduced agricultural species (i.e., peach, Asian plums), and their more sporadic hosts are native (i.e., blueberry, crabapple) (Jenkins et al., 2006), indicating a shift to exploit novel, domesticated hosts. This finding is not particularly surprising considering that wild hosts tend to be more chemically defended than domesticated hosts, which often trade off energetically expensive secondary metabolites for increased yields (Chen et al., 2015).

The reason *C*. *nenuphar* commonly uses an otherwise secondary host in the Mid‐Atlantic Region of the United States is not currently well understood, and it is also not known whether the individuals that oviposit in either blueberry or tree fruit are able to effectively use the alternate host. One possibility is that the presence of large blueberry‐growing operations in the area caused *C*. *nenuphar* to use the most prevalent nearby host, even if it is subpar or less preferred. However, although it is true that one state in the Mid‐Atlantic United States (New Jersey) is a large‐scale blueberry producer, seven of the top 10 blueberry‐producing states are within *C*. *nenuphar*'s range (Lampasona et al., 2020), and thus, their opportunity for utilizing blueberry as a host is not limited to the Mid‐Atlantic. *Conotrachelus nenuphar* also consists of two distinct strains, namely, a “northern” univoltine strain and a southern “multivoltine” strain. New Jersey populations are almost entirely the southern strain (A. L. Nielsen, unpublished data), but other regions with the southern strain do not report *C*. *nenuphar* as a significant pest of Ericaceous hosts.

The existence of this narrow‐ranged blueberry‐utilizing *C*. *nenuphar* population raises some questions we hope to elucidate in this study; namely, are *C*. *nenuphar* adults that are collected from either blueberries or peaches likely to utilize a novel host as oviposition sites? Also, assuming they do oviposit on the novel host, would their larvae experience fitness costs in the novel host compared with their collected host? To answer these questions, we tested for the presence or absence of host fidelity in *C*. *nenuphar* by studying the (1) attraction of adults to host plant odors of the collected and novel hosts, (2) oviposition preference of adults on the collected and novel hosts, and (3) fitness of larvae reared on hosts from where the previous generation was collected and on novel hosts.

## 2 MATERIALS AND METHODS

### 2.1 Insect and fruit sources

In early spring of 2019 and 2021, overwintered *C*. *nenuphar* adults utilizing peach as their host were collected from peach orchards at the Rutgers Agricultural Research and Extension Center (RAREC) (latitude 39°31′7.99″N, longitude 75°12′21.99″W) in Bridgeton, New Jersey (USA) (Figure 1b). Peach orchards were located in an ecosystem largely consisting of managed agricultural land (primarily apple, peach, soy, and corn), deciduous forest, and hedgerows. Surrounding forest edges were home to several Rosaceous hosts such as crabapple and wild cherry, as well as wild blueberry, potential wild hosts of *C*. *nenuphar* (Maier, 1990). Similarly, overwintered *C*. *nenuphar* adults utilizing blueberries as their host were collected from blueberry fields at an organic blueberry farm in Hammonton, New Jersey (USA) (latitude 39°39′37.53″N, longitude 74°45′14.75″W) (Figure 1b). These blueberry fields were located in the New Jersey Pinelands National Reserve, an environment dominated by several species of pine trees. Wild blueberry, huckleberry, and wild cherry occur in the forested areas adjacent to the crop plantings and could potentially be used as wild hosts by *C*. *nenuphar*. Overall, the area surrounding the blueberry fields contained mostly Ericaceous wild hosts and other blueberry plantings. The peach and blueberry sites were separated by 41.39 km (Figure 1b).

*Conotrachelus nenuphar*adults collected from these sites were used for all the following experiments. The collected adults were exclusively fed on the host plant of their origin. As a result, we collected adults from two populations with distinct origins (peach or blueberry). We chose to collect feral adults rather than adults reared from the laboratory because we were interested in the host preferences of the overwintered *C*. *nenuphar* adults, which would be difficult to produce under laboratory conditions. Overwintered adults are most ecologically relevant to our study than later generations because of their movement into the crop, which indicates that these adults make critical foraging decisions when choosing a host plant. All insects were collected using beat sheets or unbaited trunk traps (Lampasona et al., 2020), and kept in incubators at 25 ± 1°C, 70 ± 10% relative humidity, and 16:8‐h light:dark cycle until used. Adult age was indeterminate since all insects were field‐collected, but based on the timing of captures, most insects were likely to be of the overwintered generation, and thus eclosed the previous year.

All peach and blueberry flowers and fruits used for rearing insects and in experiments were collected from RAREC and the organic blueberry farm, as they were not sprayed with conventional insecticides. All samples were collected during the week of experiments.

### 2.2 Olfactory preference of *Conotrachelus nenuphar*

In 2019 and 2020, we collected 30 male and 30 female *C*. *nenuphar* adults from peaches and 30 male and 30 female *C*. *nenuphar* adults from blueberries from our two New Jersey locations (see above), for a total of 60 individuals for each sex and host plant. Collected insects were placed in incubators under the conditions described above for at least 72 h before olfactory trials began. Insects were additionally subjected to a 24‐h starvation period with no food and only distilled water prior to testing.

**FIGURE 2:**
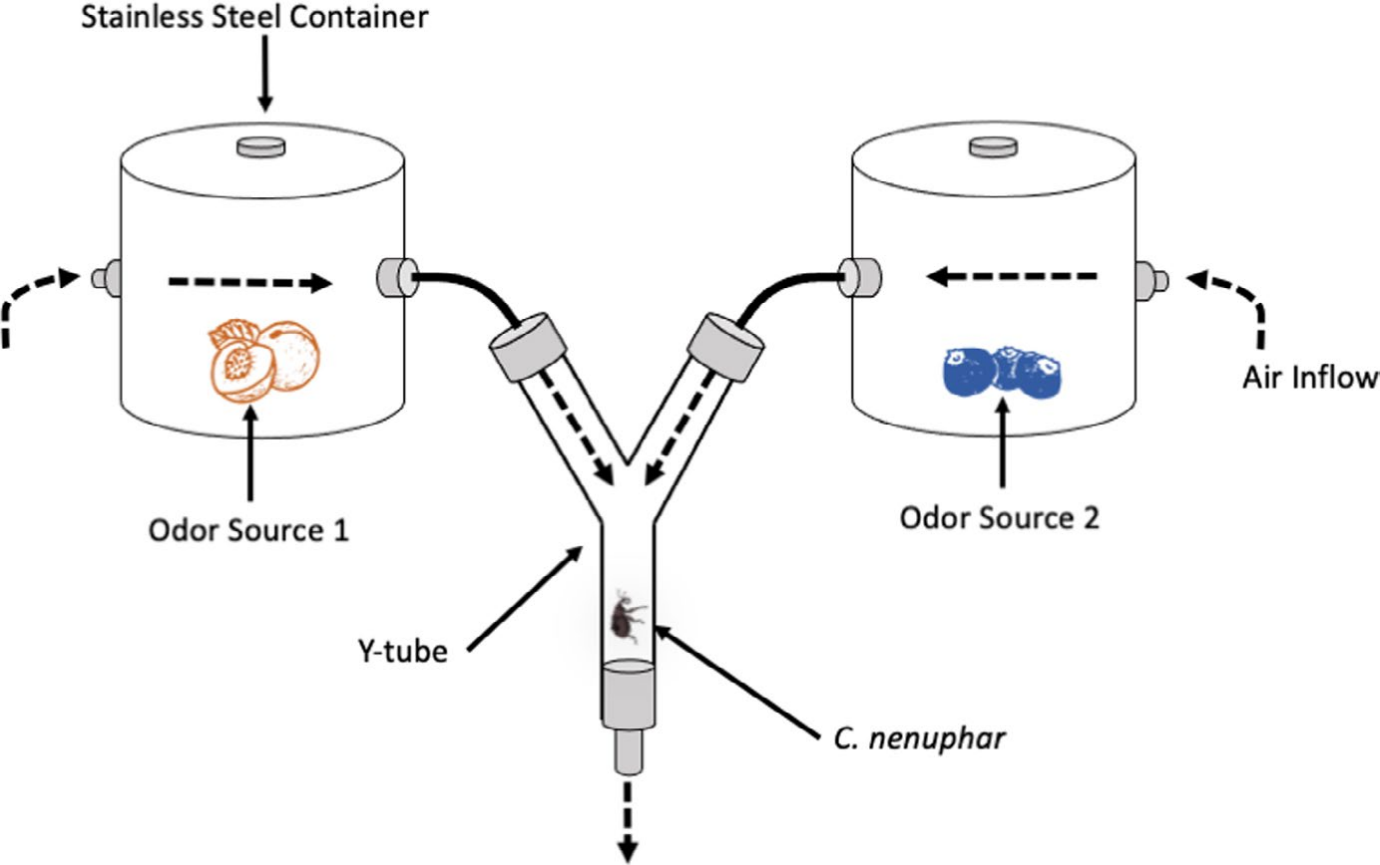
Schematic diagram of the Y‐tube olfactometer, showing full assembly and odor source placement. Air was pumped from a gas cylinder through the stainless containers and into the two arms of the olfactometer. The Y‐tube was 40 mm in diameter × 36 cm long

Olfactory bioassays were conducted in a 40‐mm‐diameter × 36‐cm‐long glass Y‐tube olfactometer that had a 50° inside angle (Sigma Scientific LLC) (Figure 2). The Y‐tube was placed in a particleboard box in a darkroom lit only with a 20‐W red LED light and maintained at approximately 25° during the insect's scotophase. Incoming laboratory‐grade air (Airgas Company) was pushed through one of two customized 4.5‐L stainless steel crock pots, each with two openings allowing air to flow in and out of a glass chamber (Figure 2). Each pot held fresh cuttings of either peach or blueberry plants (odor source). Each odor source consisted of 300 g of flowers and leaves or 600 g of fruit and leaves (all leaves collected from the same plant as flowers or fruit) for each testing period and subsequently connected by tubing to an arm of the Y‐tube. The airflow was modulated by an inline flowmeter (Gilmont Instr., Barnant Co.) set to 12 L/min to deliver 6 L/min to the olfactometer arm. Glass components of the Y‐tube were cleaned with 70% ethanol and air‐dried between each replicate, and the left/right position of each basin was swapped to mitigate potential directional bias. Individual *C*. *nenuphar* adults were transferred using featherweight forceps and placed in the Y‐tube specimen adapter, which was then attached to the Y‐tube. After attachment, a stopwatch was started. If the insect spent 60 s in either arm, or after 12 min had elapsed (whichever came first), the timer was stopped. If the insect spent 60 s in an arm, it was considered a “choice” for the odor proximal to that arm and was recorded as such. If after 12 min the insect did not spend 60 s in either arm, the insect was placed in a “no‐choice” category and removed. Insects that did not make a choice were excluded from the statistical analysis. All individuals were used only once for an experiment, and new plant material was used for each experiment.

### 2.3 Oviposition preference of *Conotrachelus nenuphar*

In 2020, we used fresh fruit to test for *C*. *nenuphar* oviposition preference between peach and blueberry in the laboratory. Small sections of peach and blueberry branches were thinned to one peach or five blueberries free of visible insect damage. Each branch section was then cut, inserted into soaked floral foam, and placed inside a mesh of 30.5 × 30.5 × 30.5‐cm insect rearing cage (BioQuip Products Inc.). In each cage, we alternated the left/right placement of the fruit between an equal number of replicates to mitigate directional bias. Insects were collected from our New Jersey sites and maintained in the laboratory as described above. Twenty‐three *C*. *nenuphar* male/female pairs collected from peach, and 23 pairs collected from blueberry were placed inside the cages (one pair per cage). Each caged pair was provided with both one peach cutting and one blueberry cutting as a choice test. In addition, 10 pairs were provided only with one host as a no‐choice check and 10 extra fruit cuttings were held in cages with no insects as “untreated” controls to determine whether field oviposition had occurred but had gone unnoticed. Prior to the experiment, individual male/female pairs were given a 24‐h starvation period in microcentrifuge tubes. Afterward, *C*. *nenuphar* pairs were introduced into the cages and allowed for 48 h to oviposit freely on either host, after which all insects and fruit were removed, and the number of oviposition scars on each fruit was counted.

### 2.4 Offspring performance of *Conotrachelus nenuphar*

In 2019, we thinned 60 blueberry and 60 peach branches down in the field so that each branch held only three peaches or 10 blueberries each. These branches were covered with a sleeve netting made of 5‐gallon paint strainer bags and secured at the base of the branch to prevent wild insects from colonizing the fruit before we introduced the *C*. *nenuphar* adults. *Conotrachelus nenuphar* adults were collected from peach trees and blueberry bushes during the week of 20 May 2019, as described above, and kept in 946‐ml plastic deli containers with fruit collected from blueberry fields or peach orchards. Insects collected from peach and blueberry were not co‐mingled and were grouped into male/female pairs and placed in microcentrifuge tubes for a 24‐h starvation period. Insects were then moved to the sleeve cages on their respective outdoor hosts. Mating occurred at any point after the introduction of males and females, although since they were wild‐caught, it was possible they had already mated. As such, the inclusion of the males was only to ensure mated status during the experiment.

A total of 60 *C*. *nenuphar* adult pairs collected from blueberry were individually placed on blueberry branches (1 pair per branch), and 60 pairs were placed individually on peach branches. This was done over a 3‐week period using 20 pairs/week for the first 2 weeks, then 15 pairs for the third week (due to lower insect captures) for a total of 55 replicates. Insects were allowed to oviposit for 4 days inside the sleeves and then removed. Fruit was kept on the branches for an additional 48 h before removal and placement in rearing containers in the laboratory. The number of oviposition scars on each fruit was counted, and each fruit was weighed to calculate the number of eggs per gram of fruit. All containers with fruit were kept in incubators at 25°C and a 16:8‐h light: dark cycle for 60 days. As larvae emerged from the fruit, their weight and head capsule width (mm) were measured as a proxy for body mass and size, respectively. The accumulated degree‐days (DDs; using a base temperature of 10°C) between adult introduction and larval emergence were recorded for each larva during the 60‐day observation period. DDs were calculated using the following formula: Daily DD_10_ = mean daily temperature − base temperature (10°C).

### 2.5 Data analyses

All data were analyzed in JMP Pro 16 (SAS). The Y‐tube olfactometer data were analyzed using the chi‐square goodness‐of‐fit tests to determine whether *C*. *nenuphar* adults preferred one host plant odor over the other. Each group of insects (based on their respective collected host) was tested separately. Because of the natural thanatosis response of *C*. *nenuphar*, some insects will “play dead” throughout the entire trial. As such, insects that did not respond (i.e., insects that stayed in the release area or that stayed inside the main body of the olfactometer without moving into either arm for the 12‐min test duration) were not included in the statistical analysis.

Choice oviposition data were analyzed using the nonparametric Steel–Dwass all‐pairs test, as data did not meet assumptions of normality. The proportion of eggs laid on each host was compared between insects collected from peach and those collected from blueberry. No‐choice oviposition data from the larval performance study were analyzed separately using the Steel–Dwass all‐pairs tests using the same combinations as above (i.e., eggs laid on each host were compared between insects collected from peach and blueberry), since data were not normally distributed.

To assess larval performance, we calculated a fitness index based on the method used by Jallow and Zalucki (2003). The fitness index = *w* × *h* × *d* × *s*, where *w* = weight of 4th instar larvae (g), *h* = head capsule width (mm), *d* = DD_10_ accumulation until larval emergence, and *s* = survival rate from egg to larval emergence. All values in the equation were means from each of the 55 replicates.

Each fitness metric (weight, head capsule size, development time in DDs, and survival), and the overall fitness index were tested separately to determine which collected/novel host combination (i.e., blueberry/blueberry, blueberry/peach, peach/blueberry, and peach/peach) was different from each other; each combination of collected and novel host was treated as an independent variable. We analyzed these data using the nonparametric Kruskal–Wallis test after data did not meet assumptions of normality. Post hoc comparisons were made using the Dunn method for joint ranking.

## 3 RESULTS

### 3.1 Olfactory preference of *Conotrachelus nenuphar*

**FIGURE 3:**
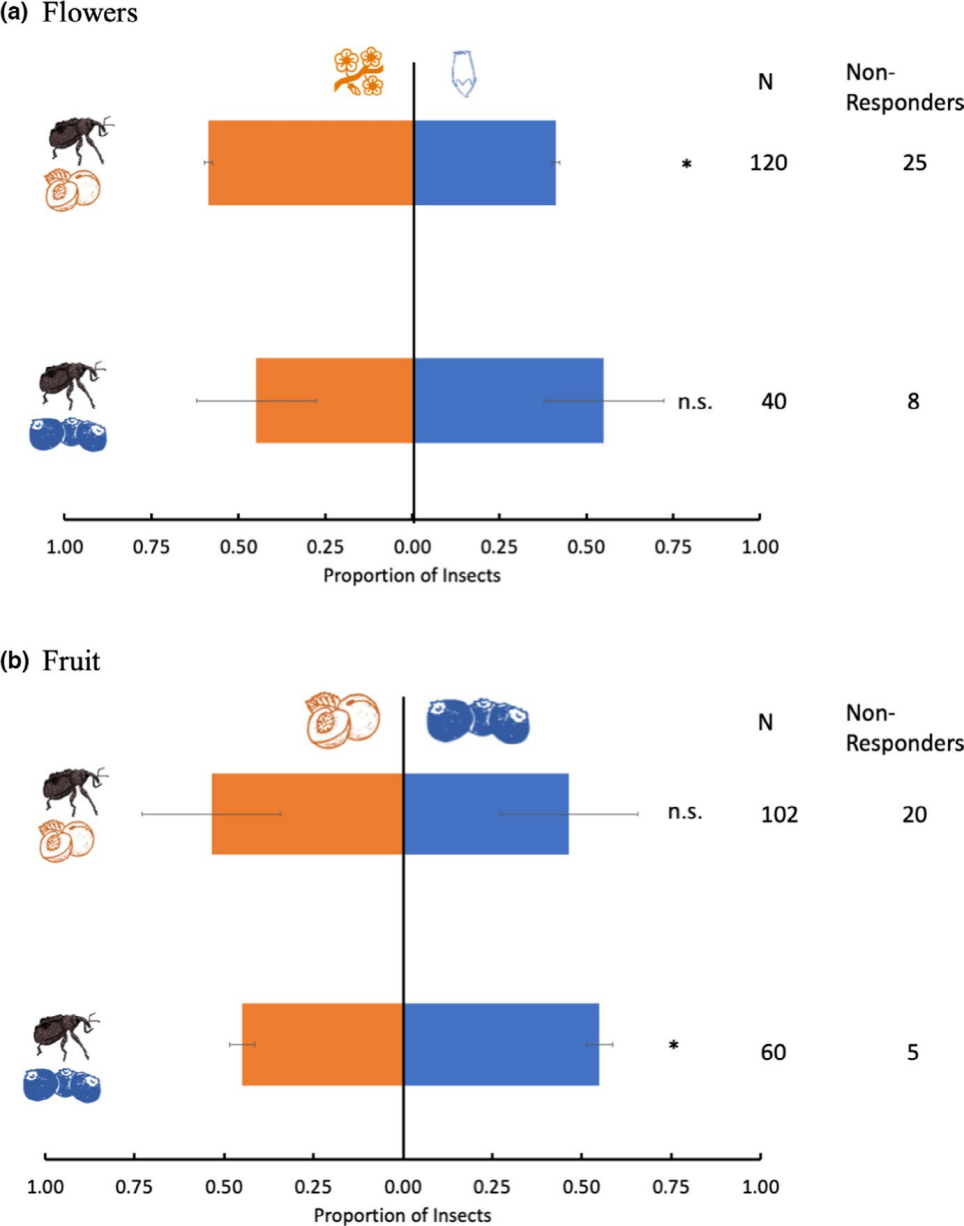
*Conotrachelus nenuphar*adult response (means ± SEM) to odors from either blueberry or peach flowers (a) and fruit (b). *Conotrachelus nenuphar* adults were collected from either blueberry fields or peach orchards in southern New Jersey (USA). Data were analyzed using the chi‐square goodness‐of‐fit tests. An asterisk (*) indicates a significant difference between treatments (*p* ≤ .05). n.s., no significant difference between treatments (*p* > .05); *N*, number of individuals tested. Nonresponders were omitted from the analyses

In Y‐tube assays, *C*. *nenuphar* collected from peach were significantly more attracted to peach flower odors than blueberry flower odors (*χ* ^2^ = 4.652, df = 1 *p* = .0312) (Figure 3a), but they did not differentiate between odors from the two fruits (*χ* ^2^ = 0.781, df = 1, *p* = .377) (Figure 3b). *Conotrachelus nenuphar* adults from blueberry were only marginally more attracted to odors of blueberry flowers than to odors from peach flowers (*χ* ^2^ = 3.6, df = 1, *p* = .058) (Figure 3a), but were significantly more attracted to the odors of blueberry fruit than to odors from peach fruit (*χ* ^2^ = 5.4, df = 1, *p* = .02) (Figure 3b). Sex had no significant effect on response in any group (*p* > .05) and was thus excluded from the data analyses.

### 3.2 Oviposition preference of *Conotrachelus nenuphar*

**FIGURE 4:**
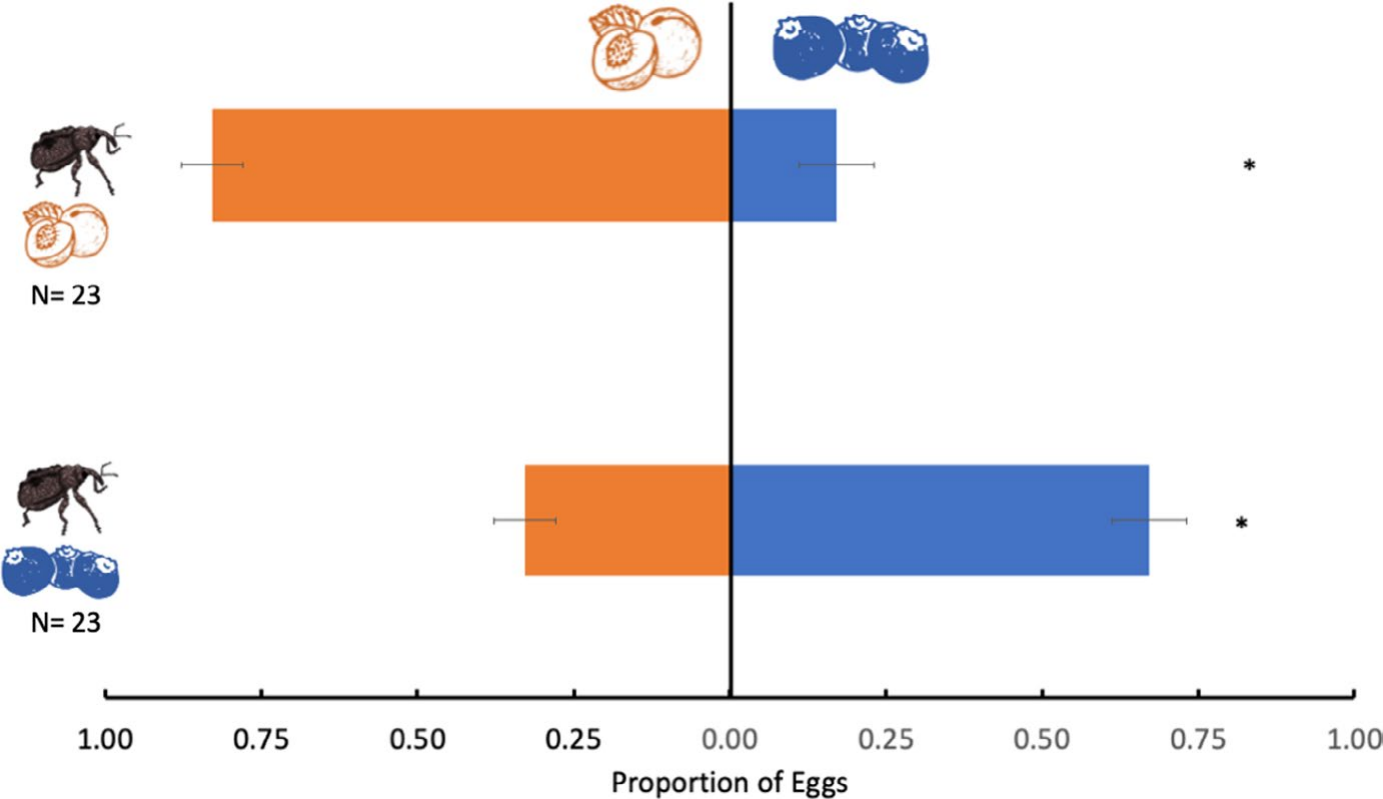
Proportion of eggs (means ± SEM) laid by *Conotrachelus nenuphar* females collected from blueberry and peach on the host they were collected from and on novel hosts in choice experiments. An asterisk (*) indicates a significant difference between treatments (*p* ≤ .05). The data were analyzed using the Steel–Dwass all‐pairs test. *N*, number of insect pairs tested

*Conotrachelus nenuphar*females from each population (blueberry or peach) disproportionately deposited eggs in their collected host when provided a choice (Figure 4). However, the difference was more pronounced among *C*. *nenuphar* from peach (*Z* = 5.262, *p* < .001), which laid 82.9% of their eggs on peaches and laid only 17% on blueberries. Although still significantly preferring their collected host, *C*. *nenuphar* females from blueberry displayed numerically less preference, laying 67.2% of their eggs on blueberries and 33% on peaches (*Z* = 3.225, *p* = .001) (Figure 4).

**FIGURE 5:**
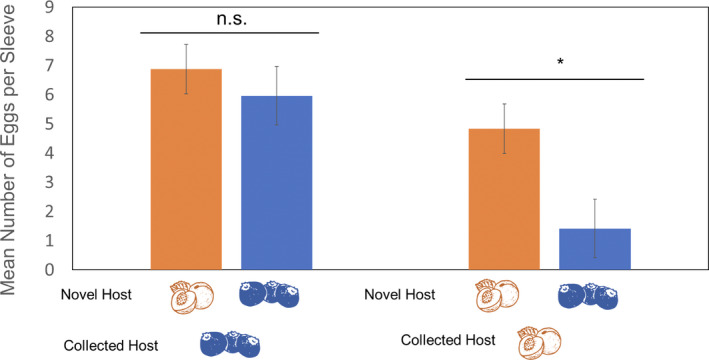
Number of eggs (means ± SEM) laid by *Conotrachelus nenuphar* females collected from blueberry and peach on the host they were collected from and on novel hosts in no‐choice experiments. Females were kept in sleeve cages and given a single oviposition choice of either peach or blueberry fruits. Data on eggs laid between novel hosts were analyzed separately for each collected host. The data were analyzed using the Steel–Dwass all‐pairs test. An asterisk (*) indicates a significant difference between treatments (*p* ≤ .05). n.s., no significant difference between treatments (*p* > .05)

In no‐choice trials, *C*. *nenuphar* females from peach laid significantly fewer eggs on blueberry than on peach (*Z* = 2.583, *p* = .0481), whereas *C*. *nenuphar* females from blueberry laid similar numbers of eggs on both hosts (*Z* = 0.459, *p* = .9678) (Figure 5).

### 3.3 Offspring performance of *Conotrachelus nenuphar*

**FIGURE 6:**
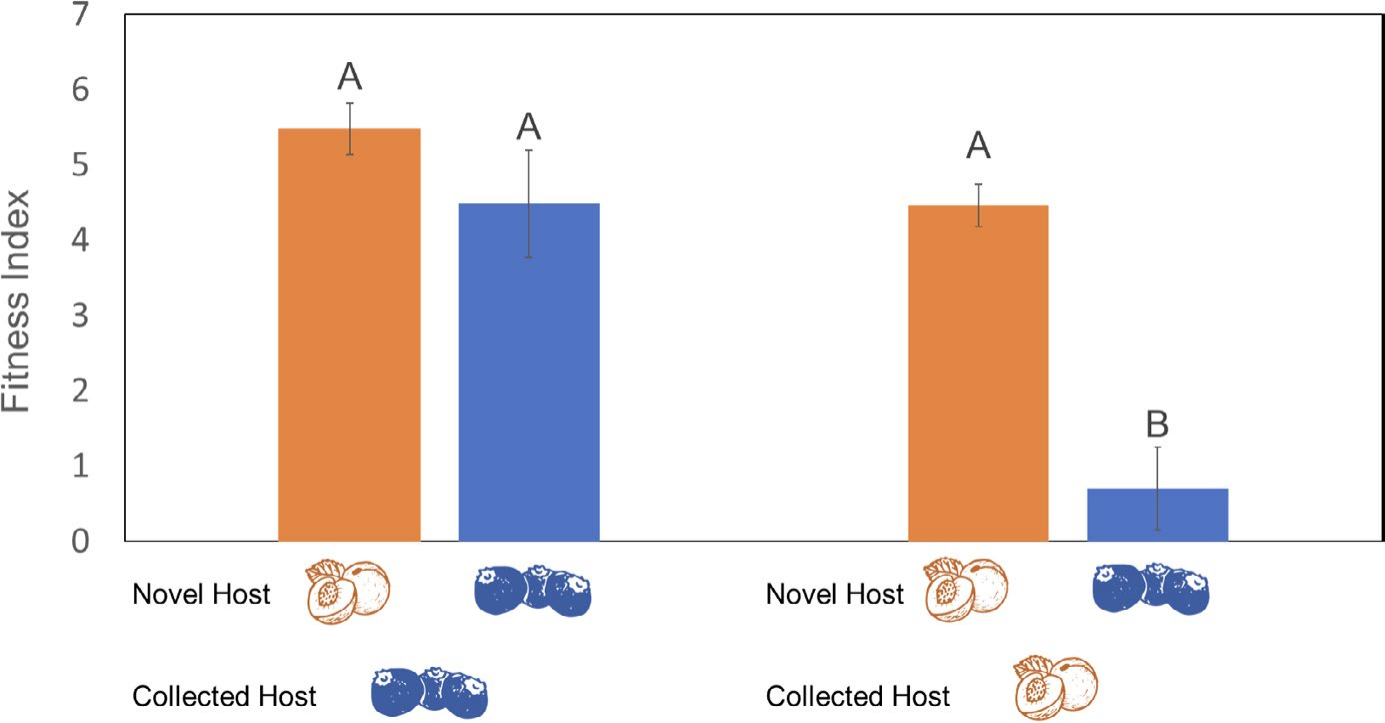
Fitness index (means ± SEM) of *Conotrachelus nenuphar* offspring of adults collected from peach or blueberry (“Collected Host”) and reared on peach and blueberry (“Novel Host”). Fitness index = *w* × *h* × *d* × *s*, where *w* = weight of 4th instar larvae (g), *h* = head capsule width (mm), *d* = DD_10_ accumulation until larval emergence, and *s* = survival rate from egg to larval emergence. Different letters indicate a significant difference between collected and novel host combinations. The data were analyzed using the Kruskal–Wallis test, with the Dunn test used for post hoc comparisons (Dunn, *α* = .05)

Analysis of larval fitness showed significant differences in fitness between different collected host/novel host combinations (*χ* ^2^ = 33.993, df = 1, *p* = <.0001). *Conotrachelus nenuphar* offspring reared on a host that was the same as the adult host exhibited high fitness (Figure 6). Also, *C*. *nenuphar* offspring of adults collected from blueberries were equally fit when reared in either peach or blueberry as novel hosts (Figure 6). However, *C*. *nenuphar* offspring of adults collected from peaches had significantly lower fitness on blueberries than on peaches (Figure 6). Although their overall fitness was similar (Figure 6), offspring of adults collected from blueberries had higher body mass and larger head capsule, and developed faster when reared in peach than in blueberry (Table 1). Larvae of insects collected from peach were heavier, developed faster, and had much higher survival when reared in peach than in blueberry (Table 1). These results indicate that peach is a better host for *C*. *nenuphar* larvae than blueberry and that offspring of adults collected from peach incur a larger fitness cost when utilizing a novel host such as blueberry than the larvae from adults collected from blueberry.

**TABLE 1:** Performance measurements (means ± SEM) of *Conotrachelus nenuphar* larvae used for the fitness index calculations

Previous generation's collected host	Novel host	Weight (mg)	Head capsule (mm)	Degree‐day accumulation^a^	Survival (%)
Blueberry	Blueberry	12.8 ± 0.2 C	0.66 ± 0.005 B	288.29 ± 3.07 A	71.9 ± 5.0 A
	Peach	18.1 ± 0.3 B	0.68 ± 0.006 A	276.91 ± 2.34 B	63.5 ± 6.0 A
Peach	Blueberry	14.1 ± 0.8 C	0.67 ± 0.03 AB	286.27 ± 10.02 AB	12.0 ± 5.0 B
	Peach	19.0 ± 0.3 A	0.64 ± 0.007 B	266.36 ± 2.78 C	56.9 ± 7.0 A

Note

Different letters within a column are significantly different from each other (Dunn test, *α* = .05).

^a^ Degree‐days were calculated using a base temperature of 10°C.

## 4 DISCUSSION

This study demonstrates that populations of the frugivorous plum curculio, *C*. *nenuphar*, exhibit differences in their preference and performance depending on whether the host plant they were collected from matches their novel host. As expected by the PPH, *C*. *nenuphar* collected from peach orchards had higher fitness on peach and lower fitness on blueberry. In contrast, *C*. *nenuphar* collected from blueberry fields displayed no significant difference in oviposition under a no‐choice scenario or in larval fitness on either host. Both *C*. *nenuphar* populations also showed higher attraction to odors from the host they were collected from than to those of a novel host.

*Conotrachelus nenuphar*females tend to prefer tree fruit to berries, and among tree fruit, they display a hierarchy of attraction, with Japanese plum being the most attractive, followed by European plum, peach, sweet cherry, tart cherry, apricot, pear, and apple (Jenkins et al., 2006; Leskey & Wright, 2007). Notably, this preference hierarchy also conforms broadly to the PPH, as apples are dense fruits that can crush larvae as they grow (Crandall, 1905; Quaintance & Jenne, 1912). By prioritizing egg laying in stone fruits, female *C*. *nenuphar* can ensure higher larval survival (Jenkins et al., 2006). Based on the literature, any *C*. *nenuphar* choosing to oviposit on a berry over a tree fruit is a noticeable break to existing host preference assumptions (Jenkins et al., 2006). In general, our data support the hypotheses that peach is a more suitable host for *C*. *nenuphar* larvae than blueberry and that females choose hosts in accordance with the PPH.

Although *C*. *nenuphar* has historically been described as a pest primarily of Rosaceae in North America (Quaintance & Jenne, 1912), they occasionally use alternative hosts as well. In some regions, these hosts include blueberry, an Ericaceous plant native to North America, with wild and cultivated genotypes occurring commonly in the *C*. *nenuphar*'s distribution range. They are occasionally observed using other Ericaceae such as deerberry and huckleberry (Jenkins et al., 2006). However, in most regions of the United States, *C*. *nenuphar* is not a significant pest of blueberry, despite their overlapping ranges (Polavarapu et al., 2004). This information would indicate that most *C*. *nenuphar* are “tree fruit‐oriented” (i.e., they generally prefer tree fruit over berries), and our data suggest that the tree fruit‐oriented individuals experience a fitness cost for their larvae on blueberries. If this cost is true for *C*. *nenuphar* populations broadly, it is likely that this general preference for tree fruit would limit their exposure to blueberries, probably in large enough numbers to avoid being considered a serious pest. Because *C*. *nenuphar* females from peach exhibit fitness costs on blueberry, it is likely that these “peach” populations are not abundant in most blueberry‐producing areas where major *C*. *nenuphar* infestations are reported.

Overall, both *C*. *nenuphar* adults from peach and blueberry that we tested exhibited host fidelity in all‐choice trials. Host fidelity can be a strong predictor of where an insect's larvae will exhibit fitness gains (Pappers et al., 2002). Females often exhibit attraction to a host they were successfully reared on, which continues the generational utilization of a successful host to the exclusion of others and potentially results in divergence that precludes the successful use of alternate hosts (phenologically or biologically) (Diepenbrock et al., 2016; Feder et al., 1994). In our study, we used overwintered *C*. *nenuphar* adults collected in the field. Future studies should be expanded to test insects reared in the laboratory on different hosts (peach and blueberry). In addition, because we used field‐collected individuals, we cannot discard that learning might have played a role in the response of *C*. *nenuphar* adults to cues associated with peach and blueberry.

The mechanism allowing *C*. *nenuphar* adults collected from blueberry to effectively utilize that host, whereas other *C*. *nenuphar* cannot, remains unknown. *Conotrachelus nenuphar* from blueberry is not known to be genetically divergent from those utilizing other hosts, and in New Jersey, both host groups (from peach and blueberry) are likely composed of the multivoltine southern strain (Crane, 2013). Nevertheless, there appear to be notable behavioral and physiological differences between populations utilizing peach or blueberry in this region. Further research, including genetic studies, will help to better explain these differences. If the blueberry‐oriented *C*. *nenuphar* populations are indeed uniquely capable of utilizing a suboptimal host compared with other *C*. *nenuphar*, these populations may become more evolutionarily distant over time, which could result in host‐race formation (Pappers et al., 2002; Pfennig et al., 2010; Via, 1999) or eventual sympatric speciation (Beltman et al., 2004; Bernays, 2001). Further studies could also elucidate whether these differences are related to plant defenses. Peach in the United States is an introduced domesticated crop, and the process of domestication can weaken plant defenses (Chen et al., 2015; Whitehead et al., 2017). Because peach is in the same genus as plum, the primary wild host of *C*. *nenuphar* (Jenkins et al., 2006), it is no surprise that *C*. *nenuphar* performs well on this and other domesticated *Prunus* species regardless of the host (Leskey & Wright, 2007). In contrast, blueberries are native plants to North America and were domesticated only recently (Mainland, 2012). Despite the possibility of domestication reducing blueberry defenses (Rodriguez‐Saona et al., 2019), wild and cultivated blueberries are rich in phenolic content and are both well defended against different feeding classes of herbivores (Hernandez‐Cumplido et al., 2018).

In summary, by testing the PPH, our study provides new insights into host use patterns of populations of a frugivorous insect pest, *C*. *nenuphar*, and the penalties they can incur when using a novel host. Understanding these preference–performance relationships for interactions between plant hosts and different herbivore populations can help provide possible explanations about why some populations of herbivores adapt to resources that can be of low quality for other populations, such as is the case for *C*. *nenuphar* populations from blueberry and peach. These studies could also explain why populations of these herbivores utilize certain resources and not others across different regions.

## CONFLICT OF INTEREST

None declared.

## AUTHOR CONTRIBUTIONS

**Timothy Lampasona:**Conceptualization (equal); Data curation (equal); Investigation (lead); Methodology (equal); Visualization (lead); Writing – original draft (equal). **Cesar Rodriguez‐Saona:** Conceptualization (equal); Investigation (equal); Methodology (equal); Project administration (equal); Supervision (equal); Validation (equal); Writing – review & editing (lead). **Anne L. Nielsen:** Formal analysis (supporting); Funding acquisition (lead); Investigation (equal); Methodology (equal); Project administration (lead); Validation (lead); Writing – original draft (supporting); Writing – review & editing (equal).

## Data Availability

The data that support the findings of this study are openly available in dryad at DOI https://doi.org/10.5061/dryad.69p8cz93j.
